# Microsporidial Stromal Keratitis in Post-Keratoplasty Eyes

**DOI:** 10.3390/jcm12113706

**Published:** 2023-05-27

**Authors:** Rossella Spena, Cristina Bovone, Nicolò Ciarmatori, Marco Pellegrini, Angeli Christy Yu, Giorgio Zauli, Massimo Busin

**Affiliations:** 1 Department of Translational Medicine, University of Ferrara, 44121 Ferrara, Italy; rossella.spena@unife.it (R.S.); nicolo.ciarmatori@unife.it (N.C.); marco.pellegrini@unife.it (M.P.); angeliyu@gmail.com (A.C.Y.); 2 Department of Ophthalmology, Ospedali Privati Forlì “Villa Igea”, 47122 Forlì, Italy; cristina.bovone@unife.it; 3 Istituto Internazionale per la Ricerca e Formazione in Oftalmologia (IRFO), 47122 Forlì, Italy; 4 Department of Environmental Sciences and Prevention, University of Ferrara, 44122 Ferrara, Italy; 5 Research Department, King Khaled Eye Specialist Hospital, Riyadh 11462, Saudi Arabia; gzauli@kkesh.med.sa

**Keywords:** corneal transplantation, deep anterior lamellar keratoplasty, *Microsporidium*, stromal keratitis, penetrating keratoplasty, post-keratoplasty infection

## Abstract

Purpose: The purpose of this paper is to report the clinical manifestations, diagnostic evaluation, management and outcomes of microsporidial keratitis in post-keratoplasty eyes. Methods: This is a retrospective review of three patients diagnosed with microsporidial stromal keratitis in post-keratoplasty eyes between January 2012 and December 2021 at a tertiary referral center (Ospedali Privati Forlì “Villa Igea”, Forlì, Italy). Results: All patients presented with fine multifocal granular infiltrates following keratoplasty for a presumed herpetic keratitis. No microorganisms were isolated from the corneal scrapings and no clinical response was observed with broad-spectrum antimicrobial therapy. In all cases, confocal microscopy demonstrated spore-like structures. The histopathologic examination of the excised corneal buttons confirmed the diagnosis of microsporidial stromal keratitis. Following therapeutic keratoplasty and treatment with an initial high dose and extended taper of topical fumagillin, clinical resolution was achieved in all eyes. The Snellen visual acuities at the final follow-up were 20/50, 20/63 and 20/32. Conclusions: Prior to definitive surgery, confocal microscopy can be employed for the in vivo detection of pathogenic microorganisms such as *Microsporidium*. In post-keratoplasty eyes, therapeutic keratoplasty and an initial high dose of topical fumagillin with extended taper can allow the resolution of microsporidial stromal keratitis with a satisfactory visual prognosis.

## 1. Introduction

*Microsporidium* is a eukaryotic obligate intracellular parasite which was once considered as a protozoa and has now been re-classified as related to fungi [1,2]. Given its intracellular nature, *Microsporidium* is difficult to isolate through microbiological cultures and tends to be resistant to antimicrobial therapy. While there have been several reports of microsporidial keratitis from endemic regions such as in Southeast and South Asia [3,4,5,6,7,8,9,10], relatively little has been published about the clinical features of microsporidial keratitis in the Western world, and even less information is available regarding the long-term outcomes of such infection following keratoplasty. An improved understanding of variable clinical presentation and disease course may increase awareness even among ophthalmologists in non-endemic areas. We report herein three cases of microsporidial stromal keratitis in post-keratoplasty eyes managed with therapeutic keratoplasty and targeted therapy with an initial high dose and extended taper of topical fumagillin at our institution.

## 2. Materials and Methods

This is a retrospective review of 3 cases of microsporidial stromal keratitis in post-keratoplasty eyes diagnosed between January 2012 and December 2021 at a tertiary referral center (Ospedali Privati Forlì “Villa Igea”, Forlì, Italy). This study adheres to the tenets of the Declaration of Helsinki and was approved by the local Ethics Committee (Comitato Etico, Ospedali Privati Forlì). Written informed consent was obtained from all patients.

## 3. Results

### 3.1. Case 1

**Figure 1:**
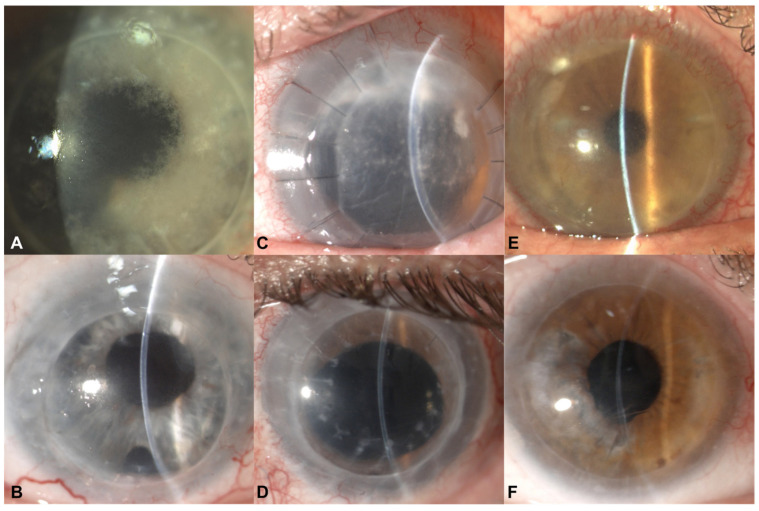
Preoperative (**A**,**C**,**E**) and postoperative (**B**,**D**,**F**) slit lamp photos of microbial stromal keratitis in post-keratoplasty eyes.

A 43-year-old male was referred for corneal infiltrates that were noted 13 months following penetrating keratoplasty for presumed herpetic keratitis. The patient denied any history of ocular trauma or systemic comorbidities. On presentation, a slit lamp exam showed white multifocal granular infiltrates within the recipient and donor corneal stroma. The overlying corneal epithelium was intact. The rest of the ocular exam was unremarkable. The microscopy and cultures of the corneal scrapings were negative. Despite intensive treatment with topical and systemic antivirals and fortified antibiotics including amphotericin-B, ceftazidime, vancomycin and amikacin, the opacities slowly coalesced and advanced centripetally (Figure 1A).

**Figure 2:**
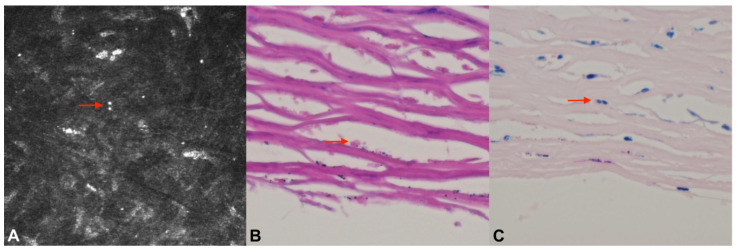
In vivo confocal microscopy (Heidelberg Retina Tomogram III-Rostock Cornea Module; HRTIII-RCM; Heidelberg Engineering GmbH, Heidelberg, Germany) demonstrates numerous hyperreflective ovoid structures within the corneal stroma (**A**). Histopathologic examination of oval bodies with dense polar staining consistent with *Microsporidium* spores with Gram (**B**) and Giemsa (**C**) staining.

The in vivo confocal microscopy (IVCM) (Heidelberg Retina Tomogram III-Rostock Cornea Module; HRTIII-RCM; Heidelberg Engineering GmbH, Heidelberg, Germany) revealed the presence of numerous hyperreflective ovoid structures, approximately 5 μm in diameter, scattered within the corneal stroma (Figure 2A). Due to a lack of clinical response, repeat therapeutic PK was subsequently performed. A histopathologic examination of the excised corneal button confirmed the presence of numerous stromal Giemsa- and Gram-positive oval bodies with dense polar staining consistent with *Microsporidium* spores (Figure 2B).

The microbiological cultures and viral PCR were negative. Topical fumagillin (2 mg/mL), which was a galenic formulation prepared by a local pharmacy for off-label use, was started every two hours and slowly tapered off over a two-year period. In the course of his care, two endothelial keratoplasties were required for graft failure following multiple episodes of endothelial rejection. Ten years after the initial assessment, the cornea was clear with no signs of recurrence (Figure 1B). The final best spectacle-corrected visual acuity was 20/50.

### 3.2. Case 2

A 52-year-old male was referred for further evaluation of a progressive infection occurring six months after a primary penetrating keratoplasty for presumed herpetic scarring on the left eye. The patient denied any history of ocular trauma or systemic comorbidities. Vision was limited to hand movement, and a slit-lamp exam revealed white deep multifocal crystalline and granular opacities extending from the superior suture tracks toward the visual axis associated with mild corneal edema and Descemet folds (Figure 1C). The IVCM showed multiple hyperreflective ovoid bodies within a network of activated keratocytes. No microorganisms were isolated from the corneal scrapings and no clinical response was observed with broad-spectrum empirical therapy with voriconazole, amphotericin-B, ceftazidime, vancomycin and amikacin for 2 weeks. The histopathologic examination of a corneal biopsy specimen demonstrated Gram- and Giemsa-positive oval-shaped spores consistent with *Microsporidium* (Figure 2C). The patient underwent a therapeutic penetrating keratoplasty, and topical fumagillin (2 mg/mL) was administered every 2 h with gradual tapering over a 2-year period. After a complete suture removal, the patient regained Snellen vision of 20/63. No evidence of recurrence was observed in the serial slit lamp and confocal microscopy examinations. Three years following the repeat penetrating keratoplasty, Descemet stripping automated endothelial keratoplasty was performed for graft failure secondary to immunologic rejection. Six years after the initial assessment, the cornea was clear, and the Snellen best spectacle-corrected visual acuity of 20/63 was maintained (Figure 1D).

### 3.3. Case 3

A 50-year-old male presented with progressive blurring of vision one month after sutureless superficial anterior lamellar keratoplasty (SALK) for presumed herpetic stromal scarring combined with cataract surgery via phacoemulsification on the left eye. The patient was maintained on topical and systemic antiviral therapy. Vision was limited to counting fingers. Fine multifocal granular infiltrates were observed within the lamellar interface (Figure 1E). Empiric broad-spectrum antibiotic therapy with voriconazole, amphotericin-B, ceftazidime, vancomycin and amikacin was initiated. The corneal infiltrates gradually enlarged and coalesced. The corneal scrapings did not reveal microorganisms on the microscopy or culture. The confocal microscopy showed multiple hyperreflective ovoid bodies within the corneal stroma. Repeat microkeratome-assisted anterior lamellar keratoplasty was subsequently performed at a deeper plane (200 um microkeratome head) than that of the initial SALK procedure (130 um). The histopathologic examination of the excised corneal button was consistent with the microsporidial spores. The microbiological cultures and viral PCR were negative. Topical fumagillin (2 mg/mL) was started and tapered off over 2 years. The serial examinations with IVCM did not show any sign of recurrence. Two episodes of stromal rejection were successfully managed medically. Four years after the initial presentation, the final visual acuity was 20/32 (Figure 1F).

## 4. Discussion

Microsporidial keratitis is a rare corneal infection which poses considerable diagnostic and therapeutic challenges. Because of the heterogeneity in clinical presentation and the absence of typical clinical features, microsporidial keratitis is not well recognized and is infrequently considered in the differential diagnosis [3,4,5,6].

Interestingly, the diagnosis of microsporidial keratitis in all cases was confounded by a common red herring—a history of presumed herpetic keratitis. While herpetic infections can be complicated by secondary infections such as microsporidial keratitis [7], it is also possible that the primary stromal keratitis may have been initially misdiagnosed as a herpetic infection. In either scenario, a high index of suspicion is warranted for cases of persistent stromal keratitis in post-keratoplasty eyes that are refractory to broad-spectrum antimicrobial therapy [8]. As previously described, microsporidial stromal keratitis can present with slowly progressive granular or crystalline-like infiltrates within the deep corneal stroma [9]. Microsporidial keratitis, although rare, should also be considered for microbial keratitis with atypical clinical features.

*Microsporidium* is a fastidious organism which cannot be grown through conventional culture media. Because it tends to involve the deep stroma, the microbiologic examination of corneal swabs and scrapings alone often fails to directly isolate the organism. Thus, histopathologic confirmation is often required for a definitive diagnosis of microsporidial stromal keratitis. Given that there are no pathognomonic signs for microsporidial infection, confocal microscopy can play a key role in the detection of pathogenic microorganisms prior to surgical intervention [10,11]. In the current series, confocal microscopy has proven to be beneficial for the detection of the spore-like structures of *Microsporidium*, which helped affirm the clinical suspicion of a non-viral pathology. Confocal microscopy has been likewise useful for the serial monitoring of treatment response postoperatively.

All cases in this series required surgical excision for definitive diagnosis and management. In terms of the surgical approach, PK has been advocated as the gold standard treatment for microsporidial stromal keratitis [3]. However, the current longitudinal study provides evidence that both deep anterior lamellar keratoplasty and penetrating keratoplasty appear to be reasonable methods for therapeutic keratoplasty depending on the extent of the disease involvement and endothelial function [12,13,14,15]. Deep anterior lamellar keratoplasty (DALK) involves the selective replacement of diseased corneal stroma while preserving the healthy unaffected endothelium [13]. DALK obviates the sight-threatening complications of open-sky surgery and does not require a prolonged postoperative course of topical corticosteroids, which, in turn, avoids secondary glaucoma. Long-term longitudinal studies demonstrated excellent cumulative survival probabilities and higher predicted endothelial cell densities after DALK compared with PK.

Since both the cumulative graft survival and median survival time decrease with every repeat PK procedure, avoiding the unnecessary replacement of host endothelium is particularly important, considering that most patients who undergo keratoplasty for infectious keratitis are at a higher risk for immune rejection and may require multiple grafts throughout their lifetime [16]. In post-PK eyes, stromal exchange can be accomplished through stromal peeling along a natural plane of separation [16,17]. Since stromal peeling exploits natural cleavage planes in post-PK eyes, the resulting tissue bed appears as a strong, homogenous and impervious layer, which is akin to the pre-Descemet’s layer–Descemet’s membrane (DM)–endothelium complex observed after type 1 big bubble formation. This results in a clear graft–host interface that is compatible with satisfactory visual outcomes [18]. Moreover, from a refractive standpoint, DALK allows the use of large-diameter grafts, which have been shown to provide superior visual outcomes without an increased risk of immune rejection and graft failure [19].

Several drugs including fumagillin, chlorhexidine, polyhexamethylene biguanide, systemic albendazole and itraconazole have been used with varying success [3,4,5,6]. Although there is still no consensus in terms of the optimal regimen and dosing, from our experience in treating this infection, an initial high dose of topical fumagillin with extended taper can result in substantially improved outcomes with no observed recurrences. While not administered systemically, topical fumagillin is well tolerated with a minimal risk of corneal toxicity [20,21].

## 5. Conclusions

This case highlights several important clinical points for the diagnosis and management of microsporidial keratitis in post-keratoplasty eyes. Such infections can present with slowly progressive stromal infiltrates with a granular crystalline-like appearance in a slit-lamp examination. Prior to definitive histopathologic examination, confocal microscopy can be employed for the in vivo detection of pathogenic microorganisms such as *Microsporidium*. While the postoperative clinical course may be marked by episodes of immunologic rejection requiring repeat surgery, therapeutic keratoplasty and initial high-dose therapy using topical fumagillin with extended taper can allow the resolution of microsporidial infection with a satisfactory visual prognosis.

## Data Availability

All data relevant to the study are included in the article.

## References

[1] Anane S., Attouchi H. (2010). Microsporidiosis: Epidemiology, clinical data and therapy. Gastroenterol. Clin. Biol..

[2] Han B., Weiss L.M. (2017). Microsporidia: Obligate intracellular pathogens within the fungal kingdom. Microbiol. Spectr..

[3] Huang H.Y., Wu C.L., Lin S.H., Lin W.C., Huang F.C., Hung J.H., Tseng S.H. (2020). Microsporidial stromal keratitis: Characterisation of clinical features, ultrastructural study by electron microscopy and efficacy of different surgical modalities. Br. J. Ophthalmol..

[4] Ang M., Mehta J.S., Mantoo S., Tan D. (2009). Deep anterior lamellar keratoplasty to treat microsporidial stromal keratitis. Cornea.

[5] Sabhapandit S., Murthy S.I., Garg P., Korwar V., Vemuganti G.K., Sharma S. (2016). Microsporidial stromal keratitis: Clinical features, unique diagnostic criteria, and treatment outcomes in a large case series. Cornea.

[6] Matoba A., Goosey J., Chévez-Barrios P. (2021). Microsporidial Stromal Keratitis: Epidemiological Features, Slit-Lamp Biomicroscopic Characteristics, and Therapy. Cornea.

[7] Mittal R., Balne P.K., Sahu S., Das S., Sharma S. (2017). Coexistence of herpes simplex virus infection in microsporidial stromal keratitis associated with granulomatous inflammation. Ind. J. Ophthalmol..

[8] Busin M., Spitznas M. (1988). Sustained gentamicin release by presoaked medicated bandage contact lenses. Ophthalmology.

[9] Nahum Y., Leon P., Ricci-Filipovic B.A., Camposampiero D., Ponzin D., Busin M. (2017). Asymptomatic Infection in Decompensated Full-Thickness Corneal Grafts Referred for Repeat Penetrating Keratoplasty. Cornea.

[10] Joseph J., Murthy S., Garg P., Sharma S. (2006). Use of different stains for microscopic evaluation of corneal scrapings for diagnosis of microsporidial keratitis. J. Clin. Microbiol..

[11] Sagoo M.S., Mehta J.S., Hau S., Irion L.D., Curry A., Bonshek R.E., Tuft S.J. (2007). Microsporidium stromal keratitis: In vivo confocal findings. Cornea.

[12] Busin M., Leon P., Nahum Y., Scorcia V. (2017). Large (9 mm) Deep Anterior Lamellar Keratoplasty with Clearance of a 6-mm Optical Zone Optimizes Outcomes of Keratoconus Surgery. Ophthalmology.

[13] Yu A.C., Spena R., Pellegrini M., Bovone C., Busin M. (2022). Deep Anterior Lamellar Keratoplasty: Current Status and Future Directions. Cornea.

[14] Busin M., Arffa R.C., Zambianchi L., Lamberti G., Sebastiani A. (2001). Effect of hinged lamellar keratotomy on postkeratoplasty eyes. Ophthalmology.

[15] Busin M., Halliday B.L., Arffa R.C., McDonald M.B., Kaufman H.E. (1986). Precarved lyophilized tissue for lamellar keratoplasty in recurrent pterygium. Am. J. Ophthalmol..

[16] Bovone C., Nahum Y., Scorcia V., Giannaccare G., Spena R., Myerscough J., Yu A.C., Busin M. (2022). Stromal peeling for deep anterior lamellar keratoplasty in post-penetrating keratoplasty eyes. Br. J. Ophthalmol..

[17] Scorcia V., Giannaccare G., Pellegrini M., Camposampiero D., Ponzin D., Yu A.C., Busin M. (2023). Stromal peeling for deep anterior lamellar keratoplasty in a post-penetrating keratoplasty eye with hematocornea. Am. J. Ophthalmol. Case Rep..

[18] Busin M., Bovone C., Scorcia V., Rimondi E., Nahum Y., Myerscough J., Yu A.C. (2021). Ultrastructural Alterations of Grafted Corneal Buttons: The Anatomic Basis for Stromal Peeling Along a Natural Plane of Separation. Am. J. Ophthalmol..

[19] Lucisano A., Lionetti G., Yu A.C., Giannaccare G., D’Angelo S., Busin M., Scorcia V. (2022). Outcomes of Conventional 8.0-mm Versus Large 9.0-mm Diameter Deep Anterior Lamellar Keratoplasty for Keratoconus. Cornea.

[20] Didier E.S., Maddry J.A., Kwong C.D., Green L.C., Snowden K.F., Shadduk J.A. (1998). Screening of compounds for antimicrosporidial activity in vitro. Folia Parasitol..

[21] Moshirfar M., Somani S.N., Shmunes K.M., Espandar L., Gokhale N.S., Ronquillo Y.C., Hoopes P.C. (2020). A Narrative Review of Microsporidial Infections of the Cornea. Ophthalmol. Ther..

